# Targeted Hotspot Mutation Analysis of KRAS, TP53, and PTEN in Endometrial Cancer Revealed by Next-Generation Sequencing

**DOI:** 10.3390/curroncol33100601

**Published:** 2026-10-06

**Authors:** Salar Saadi Hussain, Zahra Abdulqader Amin

**Affiliations:** 1 Department of Basic Sciences, College of Nursing, Hawler Medical University, Erbil 44001, Iraq; 2 Department of Clinical Analysis, College of Pharmacy, Hawler Medical University, Erbil 44001, Iraq; zahraa.alnajaar@hmu.edu.krd

**Keywords:** endometrial cancer, KRAS, TP53, PTEN, microsatellite instability, CA1250

## Abstract

Endometrial cancer (EC) is a heterogeneous malignancy characterized by diverse molecular and clinicopathological features. This study investigated somatic mutations in three hotspot-region genes commonly implicated in EC pathogenesis (KRAS, TP53, PTEN) and their associations with clinical characteristics and biomarkers, rather than comprehensive genomic profiling. Targeted next-generation sequencing (NGS) was used to analyze hotspot regions of KRAS (exons 2 and 4), TP53 (exons 4 and 7), and PTEN (exon 2), while mutation parameters, including mutant allele frequency (MAF), allelic depth, and variant frequency, were assessed alongside clinicopathological data. Pathogenic KRAS missense mutations (p.G12D and p.A146V), TP53 missense (p.G245C) and stop-gain (p.W91*) variants, and a pathogenic PTEN mutation (p.D52Y) were identified, with MAF values ranging from 36.2% to 77.1%. One patient also exhibited microsatellite instability-high (MSI-H) status. Serum CA125 concentrations were significantly higher in serous carcinoma than in endometrioid carcinoma and non-malignant cases, suggesting potential prognostic rather than diagnostic value. This suggests an association between CA125 levels and histological subtype rather than diagnostic or prognostic value, as this study did not evaluate survival, recurrence, or longitudinal outcomes. The detected mutations highlight disruptions in estrogen signaling, DNA damage response, and PI3K/AKT pathway regulation, illustrating the diversity of alterations detectable within these three hotspot regions in EC. These findings provide insight into the mutational status of KRAS, TP53, and PTEN hotspot regions in endometrial cancer and support the potential use of identified genetic alterations as biomarkers for precision medicine and targeted therapeutic strategies, while indicating that CA125’s clinical utility in EC, whether diagnostic or prognostic, remains to be established through outcome-based studies. This also reinforces CA125’s limited diagnostic but possible prognostic role in EC management.

## Simple Summary

Endometrial cancer displays considerable genetic heterogeneity, and these differences can shape how the disease develops and responds to treatment. Next-generation sequencing in this study revealed mutations in KRAS, TP53, and PTEN, several of which are likely pathogenic, and one patient’s tumor showed high microsatellite instability (MSI-H). Interestingly, CA125 levels were elevated in serous carcinoma relative to the other histological groups, hinting that this marker may be more valuable for prognosis than for diagnosis. Together, these observations add to what we know about the molecular makeup of endometrial cancer and could eventually help guide more individualized diagnostic and treatment strategies.

## 1. Introduction

Endometrial cancer (EC) has become the sixth most common cancer in terms of incidence among women worldwide. Over 400,000 women are diagnosed with EC every year, and its incidence and mortality rates are constantly increasing [1]. The United States leads in adenocarcinoma of the endometrium, also known as EC or uterine/endometrial carcinoma, which is the most common cancer of the female genital tract [2]. Meanwhile, the European region knows it as the fourth-highest female malignancy there [3]. The incidence is especially high in highly and moderately developed countries.

Most cases are localized at diagnosis, which is often associated with a good prognosis and a 5-year survival rate of approximately 95%. On the other hand, recurrence is less responsive to subsequent treatments, with significantly poorer prognoses following relapse [4]. The disease most commonly occurs in postmenopausal females, with the mean age at diagnosis being around 60 years, although some cases are also diagnosed before the age of 45 [5]. The key risk factors are obesity, hypertension, hyperinsulinism, and prolonged exposure to unopposed estrogen, such as in polycystic ovary syndrome or infertility treatment with tamoxifen [6,7]. Obesity, hypertension, and diabetes are collectively known as the EC triad [8].

Diagnostic and prognostic strategies for EC have improved significantly. The most common approach relies on histopathologic features, including lymphovascular space invasion, histologic type, grade, and stage, to guide treatment selection and prognosis. Cancer Genome Atlas Research Network has provided a new integrated molecular classification of EC, which was associated with distinct clinical-biological features and different prognostic outcomes. This classification was based on TP53 mutation and PTEN deletion status, somatic copy number alterations, and tumor mutational burden [9]. This finding highlighted the limitations of histopathology in capturing the condition’s biological heterogeneity and opened the door to molecular tumor classification as an alternative to conventional diagnostic tests [10].

Before the current epoch of molecular characterization, EC was broadly classified into two histopathological types: type I and type II. The former is estrogen-dependent, typically low-grade, frequently encountered, and associated with a better prognosis than the latter. Type I tumors mainly consist of grade I–II endometrioid adenocarcinoma. In turn, type II carcinomas are estrogen-independent, high-grade, relatively rare, and exhibit a poor clinical outcome. This category encompasses grade III endometrioid adenocarcinoma, serous and clear cell carcinomas, undifferentiated carcinomas, and carcinosarcomas [11]. At the molecular level, this distinction between the two types of endometrial cancer (EC) is further characterized by differences in somatic mutations. First, PTEN mutations are more common in Type I tumors, whereas TP53 mutations are more common in Type II tumors. Secondly, microsatellite instability caused by DNA mismatch repair gene defects is also considered a defining feature of EC, especially in Type I [12].

On the diagnostic front, serum CA125 is the most common tumor marker used for early detection of ovarian cancer. It has been found to have poor specificity and is often elevated in non-cancerous conditions. Cytological examination of urine and vaginal lavage fluid has also been used, although it has proven to be less effective due to collection problems [13]. Current standard diagnostic tests such as transvaginal ultrasound, fractional dilation and curettage, and outpatient hysteroscopic biopsy have high sensitivity but low specificity and can be associated with patient discomfort. Newer genetic tests using NGS could improve molecular subtyping of endometrial cancer by providing greater genetic detail than traditional immunohistochemistry and polymerase chain reaction techniques. Meanwhile, researchers are developing a simplified next-generation sequencing assay that could provide a more efficient and cost-effective approach to classifying endometrial cancer subtypes [9].

Building on this rationale, the study uses state-of-the-art sequencing technology to identify somatic mutations in EC patients and classify them into clinically relevant molecular subgroups, while exploring the prognostic value of the commonly used serum tumor marker CA125 and other clinicopathological characteristics.

## 2. Materials and Methods

### 2.1. Sample Collection

This case–control study involved patients diagnosed with endometrial cancer confirmed by histopathological tests, without previous radiation or chemotherapy, who gave informed consent for participation in the study and agreed to donate samples. Of the 70 participants enrolled, 41 had histologically confirmed EC (38 endometrioid, 3 serous carcinoma) and were designated as cases, while 29 patients with histologically confirmed non-malignant endometrial pathology served as the control group. We collected socio-demographic characteristics, family history, and treatment information during personal meetings.

Formalin-fixed, paraffin-embedded tissues with histologically proven EC were retrospectively collected from Maternity Teaching Hospital and Zhin International Hospital in Erbil City. We extracted clinical information, including histopathological subtypes and tumor stage, from private labs (Al-Mufti and Loui Laboratories). We FIGO-staged the patients and performed molecular typing. We also evaluated clinicopathological characteristics and the prognostic factors of each molecular subtype.

### 2.2. DNA Extraction Process from FFPE Samples

We extracted genomic DNA from FFPE tissue samples using the QIAamp^®^ DNA FFPE Tissue Kit (Qiagen, Hilden, Germany), following the manufacturer’s protocol. We first deparaffinized sections in xylene, then rehydrated them through a series of ethanol washes to remove residual xylene. We lysed cells and removed protein by treating the samples with Buffer ATL and Proteinase K, incubating at 56 °C and 90 °C. We then added Buffer AL and ethanol to promote protein digestion and facilitate DNA binding. We then loaded the lysates onto QIAamp MinElute columns, centrifuged to purify the DNA, and washed with AW1 buffer and ethanol. Finally, DNA was eluted in Buffer ATE, and its concentration and purity were assessed by measuring the 260/280 nm absorbance ratio.

### 2.3. Next-Generation Sequencing (NGS) Assay

The NGS assay begins with library preparation, including DNA fragmentation, end repair, A-tailing, and adapter ligation. Post-ligation cleanup uses magnetic bead purification to remove unligated adapters and short fragments. Libraries undergo limited-cycle PCR amplification for enrichment, followed by further purification and quantification steps. The target enrichment phase uses hybridization of pooled indexed libraries with a specific probe panel, along with blocking reagents and Cot DNA, to reduce off-target binding. Captured DNA–probe complexes are immobilized on streptavidin beads, then washed stringently, followed by on-bead PCR amplification to produce sufficient sequencing material. Amplified libraries are then purified and quantified. First, the MGI (MGI Tech Co., Ltd., Shenzhen, China) protocol involves heat-denaturing the double-stranded library to form single-stranded DNA, followed by ligation to form circularized strands. The remaining linear DNA is digested by enzymes, thus forming circularized single-stranded DNA. The obtained single-stranded DNAs were purified and quantified to prepare the DNA nanoballs (DNBs). Next, DNBs were formed by rolling circle amplification, producing the densely packed DNBs required by the MGI sequencer. The DNBs were quantified and loaded onto the MGI DNBSEQ-G400 (MGI Tech Co., Ltd., Shenzhen, China) using a PE150 flow cell cartridge for paired-end 150-nucleotide reads. After library preparation, we prepared reagents, loaded the instruments, and ran them automatically to complete sequencing. Major steps included enzyme- and bead-based nucleic acid purification, indexed adapter ligation to enable multiplexing, hybridization capture, and final preparation of sequencing-ready DNBs for the MGI sequencer.

### 2.4. Bioinformatics Analysis

We sequenced enriched libraries on an MGI DNBSEQ-G400 instrument using PE150-FCL cartridges (MGI Tech Co., Ltd., Shenzhen, China). We analyzed the data and interpreted the NGS results using the Invitro-tek Bioinformatics Analysis System. This system primarily identifies variants associated with germline and somatic events, including those related to the studied EC. Reads were aligned to the human reference genome (GRCh37/hg19) using BWA (v0.7.17-r1198-dirty), and variants were called using the Genome Analysis Toolkit (GATK, v4.2.6.1). We designed the somatic pipeline using standard algorithms, common bioinformatics tools, and databases of cancer-related genes (including ClinVar [release 2024-01] and dbSNP [build 155]), enabling screening for oncogenic, likely oncogenic, and other clinically relevant variants.

We assigned variant pathogenicity (Pathogenic/Likely Pathogenic/VUS/Likely Benign/Benign) according to ClinVar annotations (NCBI ClinVar, accessed January 2024), which reflect germline ACMG/AMP classification criteria. Somatic oncogenicity (Oncogenic/Likely Oncogenic) was assigned separately using the Invitro-tek system’s somatic classifier integrated with the OncoKB precision oncology knowledgebase, which evaluates functional domain locations, cancer hotspot frequency, in silico deleteriousness scores (SIFT and PolyPhen-2), and curated functional and clinical evidence. These two classifications are independent and assess different properties of a variant (germline clinical significance vs. somatic cancer-driving potential); a variant may therefore carry both labels simultaneously.

### 2.5. Statistical Analysis

We performed the statistical analysis using the Statistical Package for the Social Sciences (SPSS version 27.0 software (IBM Corp., Armonk, NY, USA)). We used frequencies and percentages to summarize categorical data, and mean and standard deviation to summarize continuous quantitative data. We assessed differences in CA125 levels among the three histological groups (endometrioid, no malignancy, serous carcinoma) using the Kruskal–Wallis test, and assessed normality with the Shapiro–Wilk test. We performed pairwise post hoc comparisons using Dunn’s test with Bonferroni correction, given the unequal and small group sizes (*n* = 3 for serous carcinoma). *p* < 0.05 was considered statistically significant. GraphPad Prism (Version 10.4.1) was also used to generate graphs.

## 3. Results

### 3.1. Demographic, Clinicopathological, and Biomarker Characteristics of the Study Population (EC Cases and Non-Malignant Controls)

Table 1 presents the demographic and pathological characteristics of patients. The full cohort comprised 70 participants: 41 EC cases (38 endometrioid, 3 serous carcinoma) and 29 non-malignant controls. The mean age was 57 ± 11 years (mean ± standard deviation), and the mean body mass index (BMI) was 32.60 ± 7.74, suggesting the study included mostly postmenopausal women with obesity. Among tumor types, the most frequent malignancy was endometrioid, encompassing 54.3%, while 41.4% of patients had no malignancy, and only 4.3% had a more aggressive serous carcinoma. Additionally, most women had moderately differentiated tumors with an average grade of 2.

Pathologically, 61% of patients demonstrated myometrial invasion of more than half the uterine wall, indicating a more advanced local spread. Lymphovascular invasion was observed in 19.5% of patients, but lymph node involvement was generally low, with 90.2% showing no node metastasis and only a few cases distributed among N1a, N2, and N2a categories. Most patients (73.2%) were diagnosed with tumor stage T1, suggesting early-stage disease was common, while advanced stages (T3 and T4) were less frequent. These findings indicate a high proportion of early-detected, moderately differentiated tumors with limited spread.

**Figure 1:**
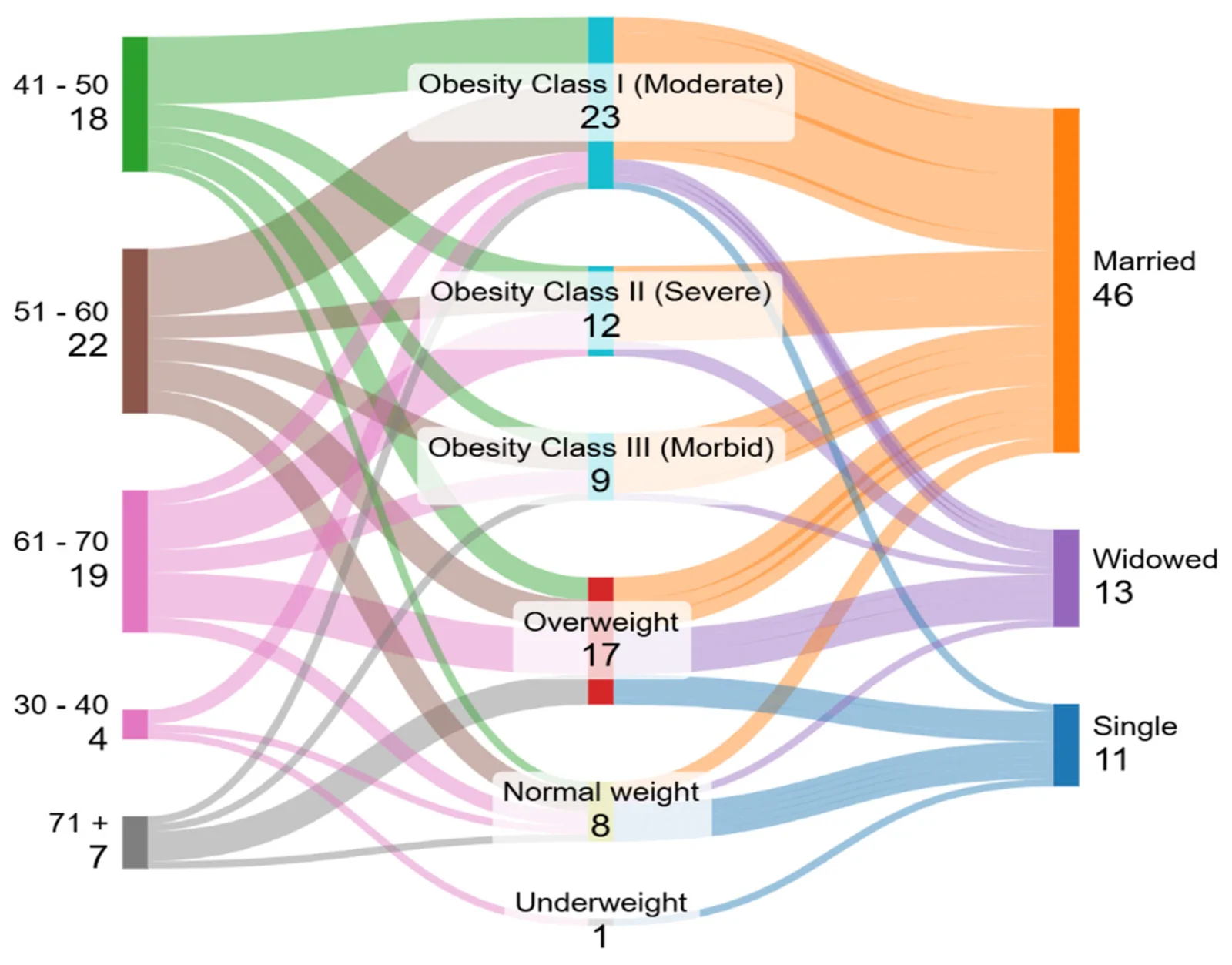
Sankey Diagram Showing the Distribution of Participants by Age Group, Weight Status, and Marital Status. Flow width and node labels indicate participant counts (*N* = 70). Diagram generated using SankeyMATIC.

Sankey diagram illustrating the flow and relationship between three key variables, such as age groups, weight status, and marital status among the participants, is shown in Figure 1. The age groups range from (30–40) years to (71 and above), and weight status categories include underweight, normal weight, overweight, and obesity classes I, II, and III. The diagram shows participants’ transitions across categories, with the width of each flow corresponding to the number of individuals in each subgroup. This allows evaluation of the distribution of weight status among selected age groups and the proportion of people with different marital statuses. The numbers in the graph indicate the number of people per age category, weight status, and marital status. For example, for the (30–40) age group, there is one underweight single, one normal weight single, and two people with Obesity Class I (Moderate), two of whom are married. These numbers reflect the size of each category, showing how many people are represented at the intersection points of the analyzed variables. It can be seen that already in the age group (30–40), the number of people with overweight or obesity is significant.

Moreover, obesity is dominated by the category of overweight people who are married. For the age group (41–50), we can see that there are 9 people with Obesity Class I (Moderate) and a small number of people with obesity classes II and III. For the (51–60) and (61–70) age groups, the number of people with overweight or obesity is the greatest. For example, for the (51–60) age group, there are 8 people with Obesity Class I (Moderate) and several people with obesity classes II and III, while for the (61–70) age group, there are several people with Obesity Class I (Moderate) and a few people with obesity classes II and III. It can also be seen that the number of widowed people prevails mainly in the higher age groups, among people with obesity classes II and III. The presented numbers allow us to evaluate the distribution of the population in these categories and see trends such as an increasing number of overweight and obese people with increasing age and differences in the distribution of marital status.

We determined CA125 concentrations among patients with different histological categories and present them in Table 2. We measured CA125 in all 70 participants: 38 with endometrioid carcinoma, 29 non-malignant controls, and 3 with serous carcinoma. The data revealed statistically significant differences between the studied groups (*p* < 0.05). A Kruskal–Wallis test revealed a statistically significant difference in CA125 levels among the three groups (H (2) = 6.886, *p* = 0.032, ε^2^ = 0.073). Specifically, the highest mean (± SD) level was observed in patients with serous carcinoma (41.87 ± 26.14 U/mL), whereas the lowest mean concentration was in the group without malignancy (17.80 ± 13.53 U/mL). Notably, among all types of tumors, the cases of endometrioid carcinoma resulted in the second-highest levels of CA125 and reached 27.19 ± 26.02 U/mL. Post hoc pairwise comparisons showed no statistically significant differences after Bonferroni correction: endometrioid vs. no malignancy, adjusted *p* = 0.163; serous carcinoma vs. no malignancy, adjusted *p* = 0.088; and endometrioid vs. serous carcinoma, adjusted *p* = 0.474. Thus, despite the high protein concentration among patients with serous carcinoma, the mean value was not considered significant because of the small sample size in this group (*n* = 3). Overall, the analysis showed that the mean (±SD) CA125 level among all participants was 23.93 ± 22.20 U/mL. The diagnostic usefulness of the CA125 test applies to malignancy, with higher concentrations observed in patients with more aggressive forms of cancer; however, its use is limited by the large variance among cancer patients and considerable overlap with benign cases.

### 3.2. Molecular Classification

Next-generation sequencing revealed somatic mutations in the three genes, KRAS, TP53, and PTEN, in the study cohort. NGS-based molecular analysis was performed on 70 samples, comprising 39 EC samples; non-malignant controls were not included in molecular profiling. Mutated exons, mutant types, and variant allele frequencies varied across these genes (Table 3, Table 4 and Table 5).

#### 3.2.1. KRAS Mutations

KRAS exon mutations were found in EC patients in exons 2 and 4 on chromosome 12. Thus, the variants found can be classified as Likely Oncogenic according to Oncogenic Classification and Pathogenic according to ClinVar. In exon 2, a specific nucleotide substitution occurs in a DNA sequence at position c.35G > A, where guanine (G) is replaced by adenine (A). This change substitutes glycine (G) at position 12 with aspartic acid (D), classifying it as a missense mutation. Such alterations can affect protein function and structure and may result in disease, as shown in Table 3 and Table 4. Thus, the variant was classified as Likely Oncogenic per the Oncogenic Classification framework and, independently, as Pathogenic per ClinVar—two separate classification systems evaluating different properties of the variant, as described in Section 2.4. In addition, Table 3 shows that MSI-H was detected among the cases, which may warrant further evaluation for mismatch-repair deficiency; mismatch-repair protein immunohistochemistry and germline testing were not performed in this case, so we could not confirm an association with Lynch syndrome. MSI-H indicates high microsatellite instability, consistent with defective DNA mismatch repair. Moreover, Table 5 illustrates the Mutant Allele Fraction of exon 2 for the KRAS gene, which was 77.10% with allelic depth and frequency (8/128, 3.72 × 10^−6^), respectively.

Other cases demonstrated that exon 4, c.437C > T, cytosine (C) at position 437 is substituted by thymine (T), leading to an alteration in amino acid A146V, a specific mutation in alanine (A) at position 146 substituted by valine (V) in the protein sequence, and considered a missense mutation; this modification can affect the protein’s structure and function, potentially leading to disease. Also, the Mutant Allele Fraction of exon 4 for the KRAS gene was 36.60%, with Allelic Depth (reference allele reads/alternate allele reads) and frequency (237/137, 0.0000031), respectively.

#### 3.2.2. Tp53 Mutations

Regarding exon mutations in the TP53 gene, specifically in exon 7 and exon 4, both are located on chromosome 17. In one case, they are classified as oncogenic under the Oncogenic Classification and as pathogenic in ClinVar. In exon 7, a specific nucleotide substitution in the DNA sequence occurs at position c.733G > T; guanine (G) is substituted by thymine (T), leading to an amino acid glycine (G) alteration at position G245C, replaced by cysteine (C), considered a missense mutation. Additionally, the mutant allele fraction in exon 7 of the TP53 gene was 36.20%, with Allelic Depth (reference allele reads/alternate allele reads) and frequency (37/21, 0), respectively, as shown in Table 3, Table 4 and Table 5. Additionally, in another case, in exon 4, a specific nucleotide substitution in a DNA sequence, where at position c.273G > A, guanine (G) is replaced by adenine (A), resulting in alteration of amino acid tryptophan (W) at position W91* to a stop codon, resulting in premature termination of protein translation. This is considered a stop-gain. The Mutant Allele Fraction of exon 4 for the TP53 gene was 67.00%, with Allelic Depth and frequency (29/59, 0), respectively.

#### 3.2.3. PTEN Mutations

Furthermore, Table 3, Table 4 and Table 5 also show that among the analyzed cases, one exhibited an exon mutation in the PTEN gene, specifically in exon 2 on chromosome 10, categorized as likely oncogenic based on Oncogenic Classification. This involved a specific nucleotide substitution in the DNA sequence, where at position c.154G > T, guanine (G) is substituted by thymine (T), resulting in the alteration of the amino acid aspartic acid (D) at position D52Y to tyrosine (Y), classified as a missense mutation. The Mutant Allele Fraction of exon 2 for the PTEN gene was 47.60%, with an Allelic Depth and frequency of 22/20 and 0, respectively.

#### 3.2.4. Oncogenic Classification Distribution

**Figure 2:**
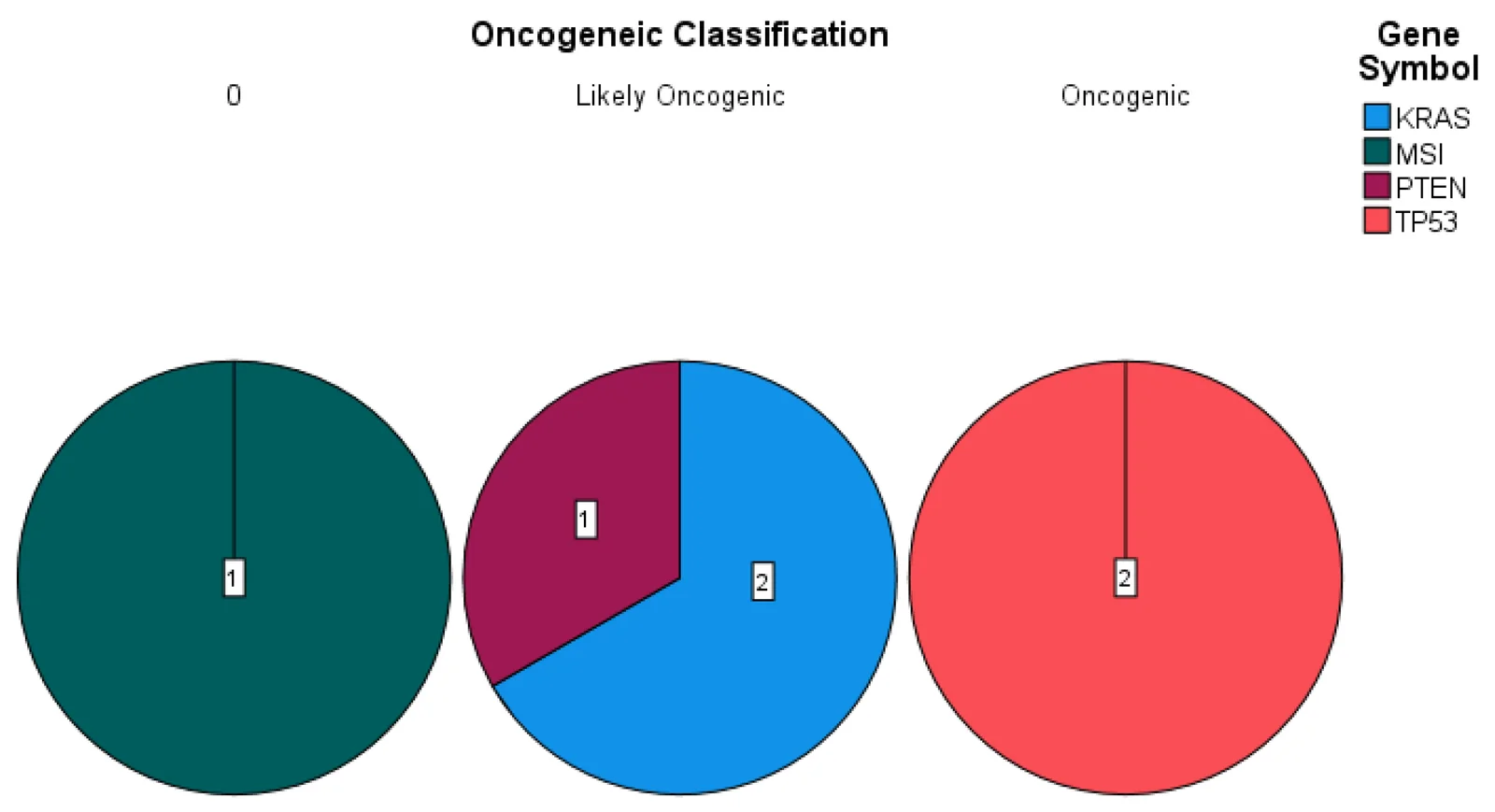
Distribution of gene mutations by oncogenic classification. Circle size and label indicate the number of alterations in each gene–classification category, based on 6 alterations (5 somatic mutations plus 1 MSI-H finding) detected among 39 sequenced EC cases.

Figure 2 illustrates the distribution of gene symbols by oncogenic classification, divided into Likely Oncogenic and Oncogenic groups. KRAS has 2 counts in the Likely Oncogenic category, while MSI has 1 count but is not categorized as oncogenic. PTEN is mentioned once in the Likely Oncogenic category, while TP53 occurs twice under the Oncogenic category. Generally, KRAS and PTEN are associated with likely oncogenic properties, whereas TP53 is considered to be oncogenic, and MSI does not appear under any category.

#### 3.2.5. Nucleotide Substitution Patterns by Chromosome

**Figure 3:**
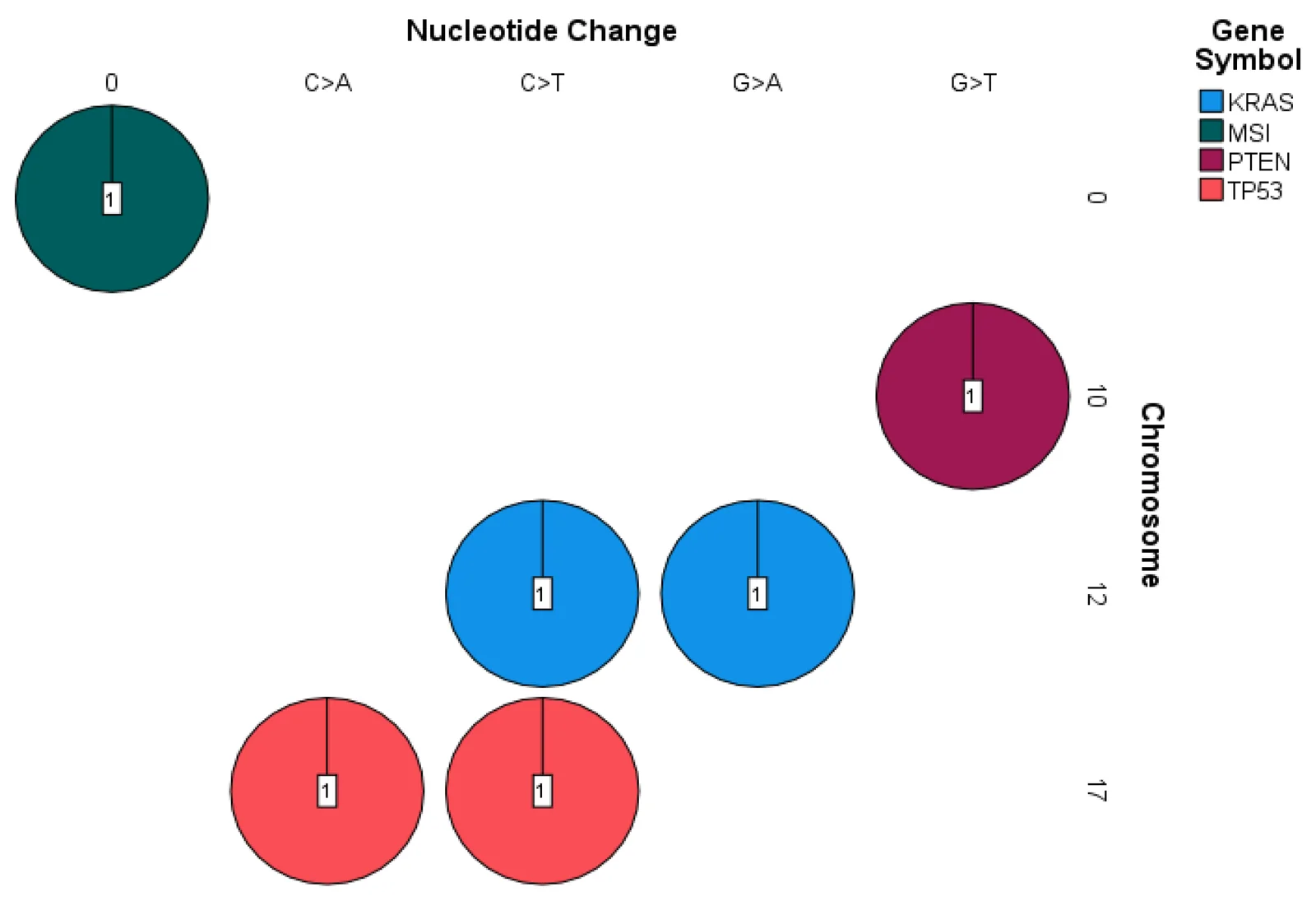
Distribution of gene mutations by chromosome and nucleotide change classification. Circle size/label indicates the number of variants at each chromosome–nucleotide-change combination (*n* = 5 somatic variants across 39 sequenced EC cases).

Figure 3 presents nucleotide variations in the mutated genes KRAS, MSI, PTEN, and TP53, along with their chromosomal locations. For each gene, the number of substitutions of each nucleotide (C > A, C > T, G > A, G > T) is given. KRAS, located on the 12th chromosome, had one C > T and one G > A change. MSI gene, which was not located on any chromosome, had one C > A mutation. On chromosome 10, PTEN had one G > T change, whereas TP53, located on chromosome 17, had one C > T and one G > A mutation. The figure shows the number of nucleotide substitutions for the genes under study.

#### 3.2.6. Nucleotide Substitution Patterns by Oncogenic Classification

**Figure 4:**
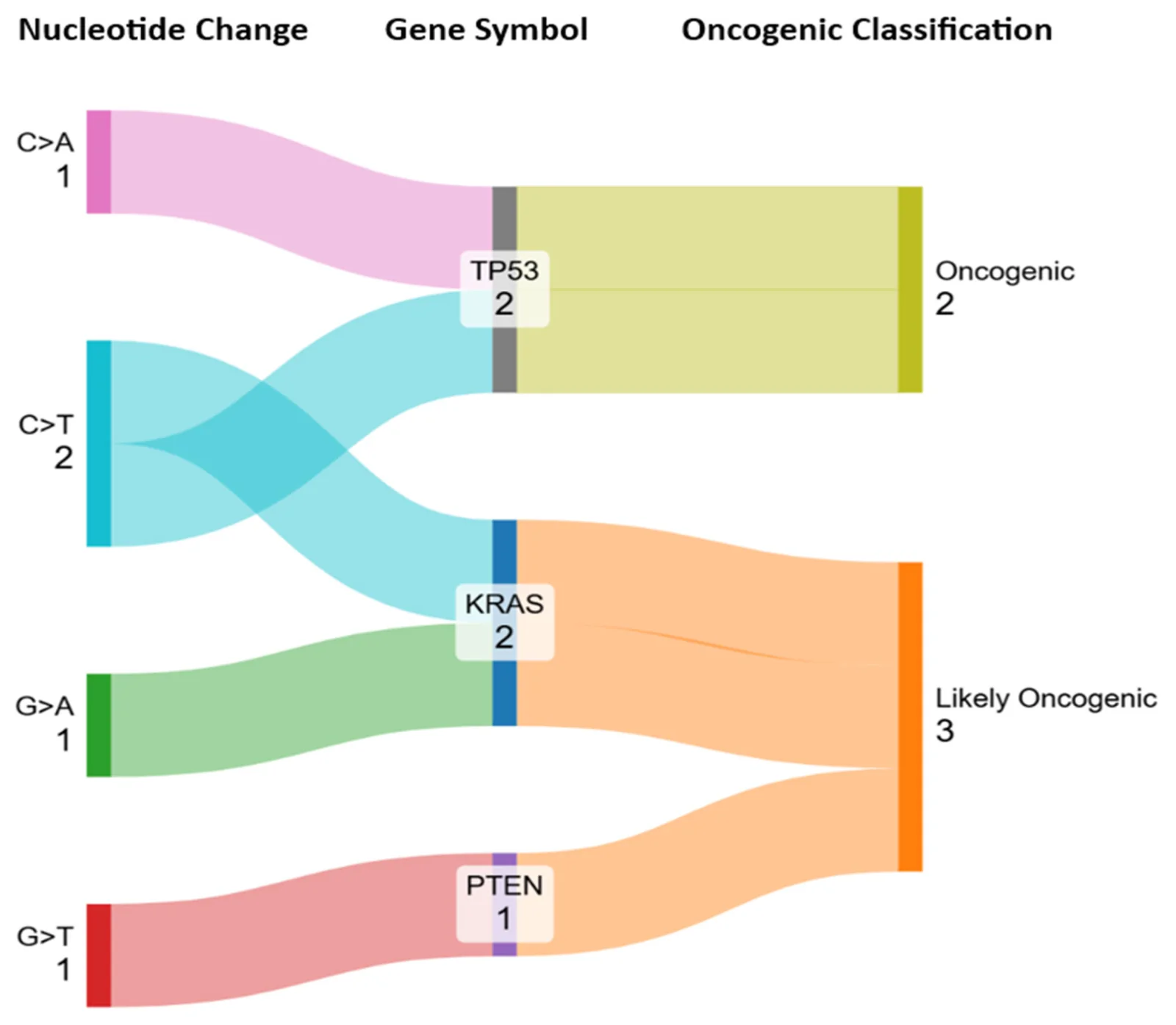
Sankey diagram showing the distribution of nucleotide changes by gene symbol and oncogenic classification. Flow width and node labels indicate the number of alterations at each nucleotide-change–gene–classification combination, based on 5 somatic mutations (TP53 *n* = 2, KRAS *n* = 2, PTEN *n* = 1) detected among 39 sequenced EC cases.

The Sankey diagram illustrates the relationships among nucleotide substitutions, affected genes, and oncogenic classification, as shown in Figure 4. We identified five alterations in TP53 (*n* = 2), KRAS (*n* = 2), and PTEN (*n* = 1). The observed nucleotide substitutions included C > T (*n* = 2), C > A (*n* = 1), G > A (*n* = 1), and G > T (*n* = 1). The two TP53 alterations involved C > A and C > T substitutions and were both classified as oncogenic. In contrast, the two KRAS alterations, involving C > T and G > A substitutions, were classified as likely oncogenic. The single PTEN alteration, characterized by a G > T substitution, was also classified as likely oncogenic. Overall, two alterations were categorized as oncogenic and three as likely oncogenic, indicating that all identified alterations in TP53, KRAS, and PTEN were oncogenic or potentially oncogenic.

#### 3.2.7. Nucleotide Changes by Patient Age in Endometrioid Histology

**Figure 5:**
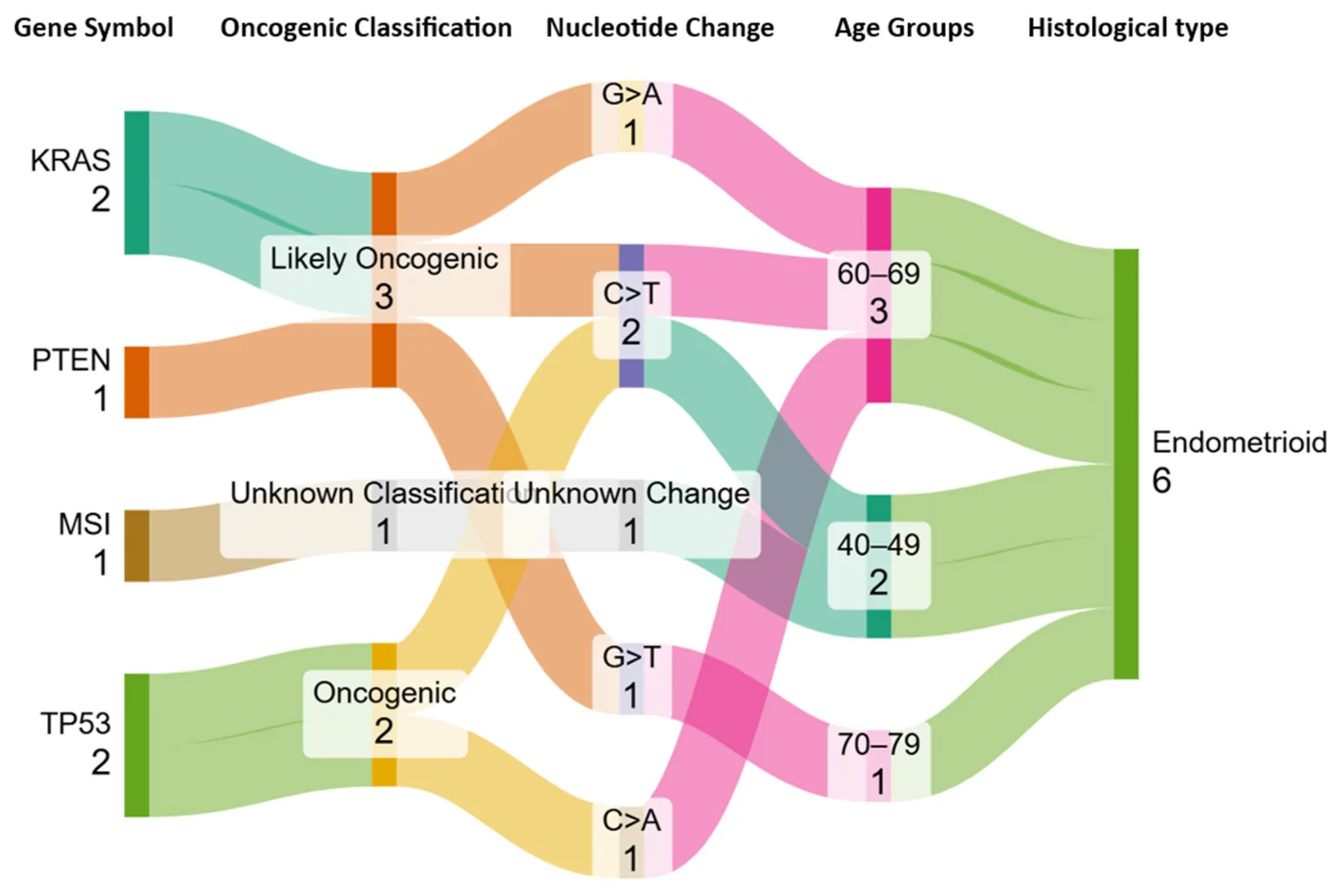
Sankey diagram showing the distribution of gene symbol, oncogenic classification, nucleotide change, age, and histological type. Flow width and node labels indicate the number of cases at each combination of variables, based on 6 altered cases (KRAS *n* = 2, TP53 *n* = 2, PTEN *n* = 1, MSI *n* = 1) among 39 sequenced EC cases, all of the endometrioid subtype.

The Sankey diagram illustrates relationships among gene alterations, oncogenic classification, nucleotide changes, patient age, and histological subtype, as shown in Figure 5. Among the detected alterations, KRAS and TP53 each accounted for two cases, whereas PTEN and MSI accounted for one case each. Three alterations were classified as likely oncogenic, two as oncogenic, and one had an unknown oncogenic classification. The observed nucleotide substitutions included C > T (*n* = 2), G > A (*n* = 1), G > T (*n* = 1), and C > A (*n* = 1), while one alteration had an unknown nucleotide change. The alterations were most frequently observed in patients aged 60–69 years (*n* = 3), followed by 40–49 years (*n* = 2) and 70–79 years (*n* = 1). All six cases were endometrioid, indicating that the molecular alterations shown in this analysis were exclusively associated with the endometrioid subtype.

## 4. Discussion

This study established relationships between demographic factors, obesity patterns, and molecular modifications in EC patients. We considered patient age, obesity, and molecular subtypes to assess disease severity. Similar to the results of epidemiological studies, obesity rates tended to increase with older ages, specifically among patients aged 51–70 years, and were predominantly observed among individuals with Obesity Classes II and III. Another study has also reported an association between obesity and increased EC risk, particularly for endometrioid cancers [14]. Earlier studies have revealed that obesity is an important risk factor for EC, associated with early-stage disease presentation and disease-specific survival in certain contexts [15]. Nevertheless, these studies have limited statistical power and control of potential confounders. Moreover, most studies on the effect of obesity on EC survival found associations with younger age, earlier disease stage at diagnosis, and isolated improvements in survival from specific causes. Endometrioid carcinomas of grade 1 or 2 were more often diagnosed at an early stage, whereas small cell carcinomas and undifferentiated carcinomas were diagnosed at a late stage [16,17].

Variations in marital status among various age groups and the high proportion of widows among the older obese population indicate the importance of considering social and lifestyle factors in the development of EC and prognosis of the patients. The study found differences in serum CA125 levels across groups. Indeed, levels were highest in those with serous carcinoma, moderate in those with endometrioid carcinoma, and lowest in those with non-malignant conditions. These results correspond with previous research that revealed elevated levels of CA125 in aggressive tumors, particularly serous carcinoma that is characterized by extensive dissemination beyond the uterus [18,19]. The large overlap between malignant and benign groups in our data suggests the limited value of using CA125 as a diagnostic criterion. Recent studies have shown that its prognostic significance outweighs its diagnostic utility, as CA125 levels are closely related to tumor stage, lymph node metastases, and patient survival rates [20,21,22]. CA125 is also not specific to EC: it can be elevated in a range of other conditions, including endometriosis, uterine fibroids, pelvic inflammatory disease, menstruation and pregnancy, and malignancies outside the endometrium (notably ovarian cancer), as well as non-gynecological conditions such as liver disease. The present study did not control for these confounding factors, which limits our ability to attribute the observed CA125 differences specifically to EC histology. The present study did not include survival, recurrence, or longitudinal outcome data, and therefore cannot independently confirm a prognostic role for CA125; our findings are limited to an association between CA125 concentration and histological subtype. The limited number of cases of serous carcinoma in our series (*n* = 3) does not allow for definitive conclusions; nevertheless, they seem to confirm the dismal natural history of this cancer subtype. Indeed, Reijnen et al., in a large prospective multicenter trial including 333 patients, found that in both low- and high-grade EC, disease-free survival (DFS) was significantly shorter in patients with CA125 levels above 35 kU/L at diagnosis. Altogether, 29.1% of patients with EC in this cohort had elevated levels [23]. Current research on CA125 in EC largely focuses on improving early detection, particularly distinguishing between patients with and without involvement of tissues outside the uterus, as well as monitoring advanced and recurrent cases [24]. Two studies have shown that CA125 has an independent predictive value for overall survival (OS), locoregional recurrence-free survival (LRFS), and disease-free survival (DFS) after adjuvant treatment for early-stage uterine cancer [25,26]. Nonetheless, these studies were single-center and retrospective, involving a limited number of cases [27]. A large single-institution study from the Czech Republic by Crha et al. further demonstrated that combining preoperative CA125 with HE4 improved stratification of regional lymph node metastatic risk in endometrial carcinoma, reinforcing CA125’s value as part of a multimarker panel rather than as a standalone diagnostic test [28].

Furthermore, in the present study, we identified mutations in the KRAS, TP53, and PTEN genes, along with MSI-H in some patients, which correspond to previously established molecular subtypes of EC from TCGA and later improvements [29]. These findings identified canonical hotspot mutations as well as less common but potentially clinically significant variants. The analysis identified the canonical G12D variant in KRAS exon 2 and the noncanonical A146V variant in exon 4. KRAS mutations were detected in 26.5% of endometrioid endometrial carcinomas [30]. Hotspot mutations affecting codons 12 and 13 are generally frequent; however, it was recently reported that substitutions at codon 146, although rare, may also lead to aberrant activation of the MAPK pathway. According to the study, KRAS mutations in EC have both prognostic and therapeutic implications, but information on noncanonical variants, such as A146V, that may drive tumorigenesis remains insufficient [31]. The observed mutant allele fraction of 36.6% in our A146V case should be interpreted with caution, as MAF from FFPE-derived targeted sequencing alone cannot establish clonality or confirm driver status; it can be influenced by tumor purity, ploidy, and sample degradation. While this variant is consistent with prior reports implicating A146V in MAPK pathway activation, functional evidence is needed to support a driver role. The observed mutant allele fraction of 36.6% in our A146V case suggests clonal representation, thereby supporting its probable role as a driver mutation. Additional functional studies are needed to determine whether these variants differentially affect sensitivity to MEK or ERK inhibition. In addition, previous findings indicate that KRAS mutation in normal endometrium may play a crucial role in adenomyosis pathogenesis [32]. KRAS has been identified as playing an early role in EC progression [33]. Another cohort showed a higher frequency of KRAS pathogenic mutations in the disease progression group [34]. The identified KRAS mutations were missense mutations (G12C, G12D, G12V, G13D, and G12S) localized within the tyrosine kinase domain, as documented by Paratore et al. [9].

TP53 mutations were detected in exons 7 and 4, including the G245C missense mutation and a W91* stop-gain. TP53 mutations indicate copy-number–high and serous-like endometrial carcinoma and strongly predict aggressive behavior [29]. The G245C variant is in the DNA-binding domain, and functional studies show it reduces transcriptional activity and enhances oncogenic phenotypes [35]. Recent EC-specific publications rarely report this variant; however, surrogate markers such as abnormal p53 immunohistochemistry have been validated as predictors of poor outcomes, even in early-stage or low-grade tumors [36]. A recent single-center cohort study from Poland similarly found that TP53-mutated endometrial cancers were associated with aggressive histopathological features, including higher tumor grade and lymphovascular space invasion, and that incorporating TP53 status upstaged nearly one-third of patients under the FIGO 2023 classification, underscoring the clinical relevance of TP53 testing beyond immunohistochemistry alone [37]. Recent data from large cohorts show that TP53 mutation status stratifies prognosis among histologic subtypes and should guide risk-adapted therapy [38]. In a previous study, three cases were observed with TP53 class III variants of uncertain clinical significance, including missense and synonymous substitutions. TP53 mutations often occur with POLE mutations or MMR deficiency, likely because impaired DNA polymerase proofreading and DNA damage repair reduce DNA replication fidelity and stability [39]. The concordance between TP53 mutation status and p53 overexpression, identified through NGS and immunohistochemistry, was reported to be 92% in endometrial cancer EC [40]. In gastric cancer, a high consistency of 90.9% was observed between TP53 missense mutations and p53 overexpression [41]. Over 51.8% (29 out of 56) of the identified mutations were observed in the high-grade serous ovarian carcinoma cohort. We also identified missense and nonsense TP53 mutations in 10 of 37 cases (27%) [42].

We identified a PTEN D52Y missense mutation in this study, consistent with PTEN’s well-established role as one of the most frequently altered tumor suppressors in endometrial cancer. Given how often it is mutated and its central role in tumorigenesis, PTEN is considered one of the more clinically significant genetic alterations in this cancer type [43]. When PTEN function is lost, the PI3K/AKT pathway becomes activated, driving changes in cell proliferation and metabolism. Notably, a TCGA-based study found that PTEN mutations tend to be associated with earlier-stage, lower-grade disease and better overall survival, yet they may also predict a poorer response to fertility-preserving treatment [44,45]. This apparent contradiction underscores why PTEN mutations require careful interpretation, with close attention to the broader clinical picture. PTEN gene mutations are a common genetic alteration in EC, found in approximately 40–80% of cases, especially in the endometrioid subtype, which is more frequent and has a more favorable prognosis than non-endometrioid types [46]. PTEN abnormalities are prevalent in all molecular subtypes of endometrioid carcinomas. Data indicate that ancillary PTEN immunohistochemistry helps diagnose endometrial neoplasms. In one study, integrating results from two panels showed that PTEN, PIK3CA, and TP53 were the most frequently altered genes in Chinese EC patients [47]. Another previous study of 115 Chinese EC patients identified PTEN (53.9%) and PIK3CA (46.1%) as the most commonly altered genes [48]. Beyond sporadic EC, PTEN alterations also underlie the hereditary PTEN Hamartoma Tumour Syndrome (PHTS); a large multicenter European cohort study reported a 10-year overall survival of 64% for PHTS-associated endometrial cancer, indicating that PTEN-related tumorigenesis in the endometrium spans both somatic and germline contexts across diverse populations [49].

In addition, PTEN has been reported to be mutated in 57–83% of EC cases, representing the most prevalent molecular alteration in early-stage endometrial cancer [34]. Furthermore, PTEN functions in the PI3K-PTEN-AKT-mTOR pathway, with somatic mutations occurring in 69–80% of endometrioid endometrial cancer EEC [50]. The activation of the PI3K pathway in patients with mutated PTEN may lead to insensitivity to progestins. Prior research has indicated a potential correlation between PTEN loss and other molecular factors, resulting in an unfavorable response to progestins [51].

Furthermore, our results identified microsatellite instability (MSI-H) in one case, suggesting mismatch repair deficiency that mismatch-repair protein immunohistochemistry or germline testing did not confirm. MSI-H ECs represent a significant TCGA-defined subtype and exhibit increased immunogenicity. Immune checkpoint inhibitors have received FDA approval for MSI-H endometrial carcinoma, rendering detection clinically relevant [52]. An earlier study found that alterations were more prevalent in MSI-H cases than in microsatellite stable (MSS) cases. However, cancer driver genes such as APC, TP53, and KRAS are frequently mutated in colorectal cancer (CRC) samples, irrespective of MSI status. The predominant mutation observed in MSI-H cases was TCF7L2 p.E384fs. Researchers have identified frameshift mutations in the TCF7L2 gene in colorectal and gastric carcinomas with high microsatellite instability [53]. Another study found MSI in 7% of ovarian clear cell carcinoma (CCC) cases. The absence of MSI in other tumor types suggests that MSI in these tumors is atypical, consistent with predominant findings in the literature. In their unselected cohort of CCC, 2% of patients were diagnosed with Lynch syndrome. The findings indicate that, alongside ovarian EC, ovarian CCC cases should also undergo routine MSI testing as part of Lynch syndrome screening to identify potential candidates for immunotherapy [54]. Additionally, other findings indicate that MSI-H EC is more frequently associated with high-grade and later-stage diagnosis compared to NSMP endometrial carcinoma [55].

Together, these findings indicate that both prevalent variants (e.g., KRAS G12D, TP53 stop-gain, PTEN loss) and less prevalent variants (e.g., KRAS A146V, TP53 G245C) are significant to EC biology. The study found that high mutant allele fractions relate to clonal driver events, while MSI-H status is associated with potential immunotherapy benefits. Including demographic risk factors such as obesity and advanced age improved prognostic value and therapeutic relevance. Future studies should clarify the role of uncommon KRAS and TP53 variants and consider molecular and lifestyle factors alongside other variables.

However, the current study has several limitations that cannot be ignored. First and foremost, the current research was conducted on a relatively small sample of patients. The problem of sample size is particularly acute for such histological subtypes of cancer as serous carcinoma (*n* = 3), given the insufficient number of cases in this group. As a result, the findings on the impact of various factors on CA125 levels may not apply to these patients. Second, although we found multiple somatic mutations in TP53, KRAS, and PTEN, and MSI-H status was associated with poor prognosis, the current study did not attempt to functionally validate these findings. In addition, the study did not account for the possible impact of lifestyle and environmental factors, such as diet, exercise, and other potential confounders. It is also necessary to note that the current study does not provide an analysis of the impact of obesity on prognosis in patients with serous ovarian cancer. Thus, the current study highlights the need for future large-scale trials with multivariable analysis to study the relationship between these variables.

## 5. Conclusions

The study provides insight into the multiparameter nature of EC based on patient age and obesity, molecular mechanisms, and serum tumor markers. The data demonstrate the importance of KRAS, TP53, PTEN mutations, and MSI-High as indicators of specific molecular genetic aberrations associated with various risk factors, many of which can be clinical manifestations of EC. In addition, the research explores the connection between CA125 levels and malignancy, as evidenced by higher levels in serous carcinoma patients than in those with benign conditions or other tumor types. Nonetheless, the lack of unique CA125 patterns suggests it is not an independent diagnostic and predictive marker for EC. Therefore, the study supports considering serum tumor markers, patient characteristics, and molecular genetic mutations when discussing EC risk and treatment options.

## Figures and Tables

**Table 1:** Demographic and Pathological Characteristics of Patients. Age, BMI, and histological type are reported for the full cohort (*N* = 70); myometrial invasion, lymphovascular invasion, lymph node status, and tumor stage are reported for EC cases only (*N* = 41).

Parameters		Mean ± SD	Frequency	%
Age (in years)		57 ± 11		
BMI		32.60 ± 7.74		
Histological type	Endometrioid		38	54.3%
	No malignancy		29	41.4%
	Serous Carcinoma		3	4.3%
Grade		2 ± 0		
Myometrial Invasion	Less than half		16	39.0%
	More than half		25	61.0%
Lymphovascular invasion	Yes		8	19.5%
	No		33	80.5%
Lymph node status	Yes		0	0.0%
	No		37	90.2%
	Yes (N2a)		1	2.4%
	Yes (N2)		1	2.4%
	Yes (N1a)		2	4.9%
Tumor stage	T1		30	73.2%
	T2		4	9.8%
	T3		6	14.6%
	T4		1	2.4%
	T5		0	0.0%
	T6		0	0.0%

**Table 2:** CA125 Levels in Patients According to the Histological Classification.

CA125	Mean	Std. Deviation	Std. Error	95% Confidence Interval for Mean		Min	Max
				Lower Bound	Upper Bound		
Endometrioid	27.191	26.018	4.221	18.639	35.743	5.280	145.200
No malignancy	17.799	13.528	2.512	12.653	22.945	4.990	49.830
Serous Carcinoma	41.867	26.140	15.092	−23.069	106.802	23.190	71.740
Total	23.929	22.197	2.653	18.636	29.222	4.990	145.200

**Table 3:** Sample-wise Gene Mutation Annotation and MSI Status.

Gene Symbol	Result/Type	MSI Status	Ensemble Gene ID	db SNP	Position (hg19)	NU Change	AA Change	Consequences
KRAS	**Positive/** **Variant**	MS-S	ENSG00000133703	rs121913529	25398284	c.35G > A	G12D	Missense
TP53		MS-S	ENSG00000141510	rs28934575	7577548	c.733G > T	G245C	Missense
.		MS-H	.	.	.	.	.	.
KRAS		MS-S	ENSG00000133703	rs1057519725	25378561	c.437C > T	A146V	Missense
PTEN		MS-S	ENSG00000171862	.	89653856	c.154G > T	D52Y	Missense
TP53		MS-S	ENSG00000141510	rs876660548	7579414	c.273G > A	W91*	Stop Gained

**Table 4:** Sample-wise Exon Mutation Annotation and Oncogenic Classification.

Exon Number	Chromosome	Ref	Alt	Nucleotide Change	Oncogenic Classification	ClinVar
Exon2	Chr12	C	T	C > T	Likely Oncogenic	Pathogenic
Exon7	Chr17	C	A	C > A	Oncogenic	Pathogenic
Exon4	Chr12	G	A	G > A	Likely Oncogenic	Pathogenic
Exon2	Chr10	G	T	G > T	Likely Oncogenic	.
Exon4	Chr17	C	T	C > T	Oncogenic	Pathogenic

**Table 5:** Per-Sample Variant Annotation and Functional Prediction. “Allelic Depth” is presented as reference allele read count/alternate allele read count.

Exon Number	Mutant Allele Fraction	Allelic Depth	Frequency	SIFT	PolyPhen2
Exon2	77.10%	38/128	0.00000372	0.01	0.257
Exon7	36.20%	37/21	0	0.0	0.996
Exon4	36.60%	237/137	0.0000031	0.0	0.802
Exon2	47.60%	22/20	0	0.001	1.0
Exon4	67.00%	29/59	0	.	.

## Data Availability

The data supporting the findings of this study are available from the corresponding author upon reasonable request. The data are not publicly available due to privacy and ethical restrictions concerning human participant information.

## Abbreviations

The following abbreviations are used in this manuscript:

**Abbreviation**	**Full Name**
AA	Amino Acid
Alt	Alternate Allele
BMI	Body Mass Index
bp	Base Pairs
CA125	Cancer Antigen 125
Chr	Chromosome
dbSNP	Single Nucleotide Polymorphism Database
DFS	Disease-Free Survival
DNA	Deoxyribonucleic Acid
DNB	DNA Nanoball
EC	Endometrial Cancer
FFPE	Formalin-Fixed Paraffin-Embedded
FIGO	International Federation of Gynecology and Obstetrics (*Fédération Internationale de Gynécologie et d’Obstétrique*)
IBM	International Business Machines
KRAS	Kirsten Rat Sarcoma Virus Oncogene Homolog
kU/L	Kilounits per Liter
LRFS	Locoregional Recurrence-Free Survival
MAF	Mutant Allele Fraction/Minor Allele Frequency
MSI	Microsatellite Instability
MSI-H	Microsatellite Instability-High
MS-S	Microsatellite Stable
NGS	Next-Generation Sequencing
NU	Nucleotide
ORCID	Open Researcher and Contributor ID
OS	Overall Survival
PCR	Polymerase Chain Reaction
PI3K/AKT	Phosphoinositide 3-Kinase/Protein Kinase B
PolyPhen2	Polymorphism Phenotyping v2
PTEN	Phosphatase and Tensin Homolog
Ref	Reference Allele
SD	Standard Deviation
SIFT	Sorting Intolerant from Tolerant
SPSS	Statistical Package for the Social Sciences
TCGA	The Cancer Genome Atlas
TP53	Tumor Protein p53
U/mL	Units per Milliliter
